# Psittacosis: An Underappreciated and Often Undiagnosed Disease

**DOI:** 10.3390/pathogens12091165

**Published:** 2023-09-15

**Authors:** Zygmunt F. Dembek, Jerry L. Mothershead, Akeisha N. Owens, Tesema Chekol, Aiguo Wu

**Affiliations:** 1 Battelle Memorial Institute, Support to DTRA Technical Reachback, Columbus, OH 43201, USA; dembek@battelle.org (Z.F.D.); tesema.chekol.ctr@mail.mil (T.C.); 2 Applied Research Associates (ARA), Support to DTRA Technical Reachback, Albuquerque, NM 87110, USA; jmothershead@ara.com; 3 Defense Threat Reduction Agency (DTRA), Fort Belvoir, VA 22060, USA; akeisha.n.owens.civ@mail.mil

**Keywords:** psittacosis, *Chlamydia psittaci*, chlamydia, Category B pathogen

## Abstract

The bacterial agent *Chlamydia psittaci*, and the resulting disease of psittacosis, is a little-known and underappreciated infectious disease by healthcare practitioners and in public health in general. *C. psittaci* infections can cause significant psittacosis outbreaks, with person-to-person transmission documented in the last decade. In this publication, we review the pathogen and its disease, as well as examine the potential for genetic manipulation in this organism to create a more deadly pathogen. Recent disease surveys indicate that currently, the highest incidences of human disease exist in Australia, Germany and the UK. We recommend the universal public health reporting of *C. psittaci* and psittacosis disease and increasing the promotion of public health awareness.

## 1. Introduction

Exposure to the bacteria *Chlamydia psittaci* may result in the disease psittacosis (also characterized as “parrot fever”). The disease is usually contracted through zoonotic transmission, and human disease often presents as atypical pneumonia in the lower respiratory tract [1]. *C. psittaci* is a US Centers for Disease Control and Prevention (CDC) Category B biological agent. As a group, Category B agents are considered to be moderately easy to disseminate, to result in moderate morbidity rates and low mortality rates, and to require specific enhancements of CDC’s diagnostic capacity and enhanced disease surveillance [2]. It is notable that psittacosis mortality was as high as 50% during the 20th century [3]. In 1929–1930, a global pandemic of psittacosis occurred, known to have affected about 800 individuals [4]. This led to a quarantine of imported parrots into the US as a preventive measure that remained in place for over 40 years [5]. In the absence of an available effective antimicrobial treatment, this disease’s impact potential could reoccur.

The United States [6], the former Soviet Union [7,8], and China [9,10] have all examined *C. psittaci* for use as a biological weapon. Rear Admiral (Ret) Ellis Zacharias stated during the 1940’s that biological weapons at that time included “bacteriological bombs containing either botulinus toxin or psittacosis virus (It should be noted that in our current understanding the term ‘virus’ was incorrectly applied in this context 76 years ago.)” and that “a single milliliter of infectious psittacosis virus could kill 20 million men” [11]. The former Soviet Union developed a psittacosis biological weapon to be used as an agricultural weapon against chicken flocks [8,12].

## 2. The Pathogen

*C. psittaci* belongs to the family Chlamydiaceae, and the order Chlamydiales. The Chlamydiaceae family is comprised of two genera: Chlamydophila and Chlamydia. The Chlamydia genus consists of 11 species, most notably *C. trachomatis*, the cause of the most common sexually transmitted infection. Recently, two novel species were discovered, *C. avium* and *C. gallinacean* [13]. *C. psittaci* is a Gram-negative, obligate intracellular bacteria containing several genotypes that can cause infection in both birds and mammals. All genotypes have host-specific preferences, yet it can be transmitted to humans, in which they cause infections primarily by colonizing human and animal mucosal surfaces [14]. The sequencing of these genotypes by genotype-specific real-time PCR supports pathogen detection [15].

All Chlamydia (including *C. psittaci*) have a biphasic developmental cycle composed of two alternating forms: a metabolically inactive elementary body (EB) and the reticulate body (RB) [16]. An important characteristic of the EB is that it is resistant to the environment and can survive outside of a host. Cell infection is mediated by the EB (0.2–0.3 μM diameter), and within-cell bacterial multiplication is mediated by the RB (0.5–1.3 μM diameter) [17]. Bacterial infection occurs when the EB attaches itself to the cytoplasmic membrane of a susceptible host cell, stimulating inclusion within a vacuole, thereby avoiding phagocytosis. The RBs cannibalize host cell ATP synthesis for cell division while inducing a host immune response. Within 48–72 h, RBs reorganize and condense to form new EBs that then depart the host cell to become available for a new infectious cycle. This unusual growth cycle helps to explain why *C. psittaci* cells will not grow in traditional bacterial culture media [18]. Furthermore, cell culture methods can take several weeks [19].

It is noteworthy that much of the research on the immune response to Chlamydia infections is from C. trachomatis, the most frequently reported sexually transmitted infection (STI) in the US [20]. In the course of its life cycle, Chlamydia evades both intracellular innate immune responses and adaptive cytotoxic T cell responses [21,22]. Chlamydial lipooligosaccharide (LOS) has a direct role in immune evasion and is conserved throughout the Chlamydia genus. Recent research indicates that *C. psittaci* inclusion membrane proteins (Incs) regulate host cell survival to enable the bacteria to evade host-cell-mediated defense mechanisms. An Inc protein subset connects with specific host factors at the endoplasmic reticulum (ER)-inclusion membrane contact site (MCS) [23]. The Incs CPSIT_0842 induces macrophage apoptosis, which may help *C. psittaci* to evade the host innate immune response and establish persistent infections [24]. Further, CPSIT_0842 upregulates the expression of IL-6 and IL-8 via Toll-like receptor (TLR) 2- and 4-mediated MAPK and NF-κB signaling pathways in human monocytes [25]. Therefore, CPSIT_0842 plays a significant role in the pathology of and immune response to *C. psittaci* infection.

## 3. *C. psittaci* Reservoirs

*C. psittaci* is a globally distributed zoonotic bacterium, and the true range of reservoirs is unknown. Most cases are identified in domesticated avian species, or domesticated or farm animals, but rare cases have been identified in a variety of zoo animals, be they birds, mammals, or reptiles. The organism has also been identified in wild birds, such as eagles and doves [26,27], and feral pigeons in urban areas are a natural reservoir of *C. psittaci* [28]. Parrots are a major host, yet prevalence and risk factors for infection in wild parrots are largely unknown. Recent research suggests there is a diverse range of novel *Chlamydiales* circulating in wildlife. Novel *C. psittaci* strains have been identified in birds that are highly virulent in humans. One study found the average wild parrot seroprevalence at 37%. Host species (including crimson rosellas (*Platycercus elegans*), galahs (*Eolophus roseicapilla*), sulfur-crested cockatoos (*Cacatua galerita*) and blue-winged parrots (*Neophema chrysostoma*)) differed in seroprevalence and *Chlamydiales* prevalence. Galahs had both highest *Chlamydiales* prevalence (55%) and seroprevalence (74%). Seroprevalence differed between sites, with a larger difference in males (range of 20–63%) than females (29–44%). This higher chlamydial prevalence than previously reported in wild parrots suggests potential reservoirs and transmission risks to humans and other avian hosts [29].

A PCR-based study of 195 North Atlantic seabirds in France revealed that 18.5% were *Chlamydiaceae* positive with two variants of a strain closely related to *C. psittaci*. The highest prevalence of bacterial shedders was found in northern gannets (*Morus bassanus*) at 41%, followed by European herring gulls (*Larus argentatus*) at 14% and common mures (*Uria aalge*) at 7% [30].

A 2013 outbreak of human psittacosis among 15 individuals (with eight hospitalizations) occurred among French workers involved in processing chickens from a mixed poultry farm (chickens and ducks). The same *omp*A genotype E/B of *C. psittaci* was isolated from both human and chicken *C. psittaci*-positive samples in this facility. This same pathogen genotype was widespread among the duck flocks which shared grassland areas with the chickens, demonstrating this pathogen’s potential to spread among commercial poultry flocks, including during their processing, and the workers involved [31].

## 4. Psittacosis in Birds and Other Animals

In birds, *C. psittaci* infection is referred to as avian chlamydiosis or ornithosis. Several Chlamydiae species may cause the disease in birds, including *C. psittaci*, *C. avium*, *C. gallinacea*, *C. buteonis*, *C. ibidis*, *C. abortus* and others [32]. It is likely underreported globally as avian disease lacks distinctive symptoms, and diagnostic tests are not readily available [33]. The US National Association of State Public Health Veterinarians recommends psittacine birds not acquired from disease-free breeding colonies receive feed containing 1% chlortetracycline (CTC) for 45 days for disease prevention [34]. The administration of antibiotics via drinking water is ineffective. The US government mandates 30 days of quarantine with CTC feed for all imported psittacine birds and advises importers to continue treatment for 15 days. Reinfection may occur, so treated birds should be isolated from untreated birds. Commercial pet bird breeding facilities can also harbor and spread *C. psittaci*, as has occurred recently in Washington state large scale breeding facility, in which 1000 birds were housed [35].

There is little in the literature concerning psittacosis in non-human mammals, although a number of wild, domesticated or farm animals have been implicated as the source of human infection. Clinical manifestation of disease is highly variable, but in general follows the protean symptoms seen in humans. In some cases, *C. psittaci* was isolated in a symptomatic animal, in other cases the animal did not evidence any symptoms of disease. The organism has been cited as the cause of placentitis in sheep, cattle, and horses, and cattle syndrome with symptoms of fever, respiratory tract infection, and a sudden drop in milk production, has been described. Similarly, dogs and cats have been infected, the variable symptoms including conjunctivitis, respiratory tract involvement, reproductive issues, and other seemingly unrelated organ systems affected [36].

## 5. Psittacosis in Humans

Although psittacosis is very common in birds, humans may contract the disease. Certain occupations are more likely to be exposed to the bacteria. These include poultry farm workers, veterinarians, bird owners, and those who work in pet stores. Although *C. psittaci* has been the primary cause of human psittacosis, the other Chlamidiae found in birds may have the potential to cause human illness. *C. gallinacea* was recently found in a population of poultry farmers, suggesting bird-to-human transmission of this species [37], and *C. abortus* strains found in birds also have potential for zoonotic transmission to humans [38]. Individuals involved with caged birds account for 70% of known cases [39]. Those who do develop disease generally present with mild symptoms or may even be asymptomatic, but certain vulnerable populations may develop severe disease or even die without treatment. Humans usually contract the disease through exposure to *C. psittaci*-containing bird droppings or secretions that have dried, producing bacteria-laden dust that can be inhaled. The bacteria may also be transmitted via bird bites.

Other sources of transmission do exist. Aerosol transmission in the laboratory in the absence of biosafety controls was first documented in 1930 [40]. An often-overlooked transmission vector are *C. psittaci*-infected sheep, cattle, and goats during the birthing season [41,42,43] due to lack of specific testing [44]. *C. psittaci* transmission to humans has recently been documented by infected equine placental material. In 2014, in New South Wales, Australia, five psittacosis cases (three probable, two suspected) were identified among veterinary staff and students who had delivered a foal, which later died, or handled the placenta. Contact with birds was not associated with illness [45]. The detection of a *C. psittaci*-6BC-like strain in equine tissue suggests that the horse may have been infected via ‘spill-over’ from an infected native Australian parrot [46]. This recently identified disease transmission vector may indicate that additional yet-to-be-discovered psittacosis exposure pathways may exist.

Person-to-person disease transmission is rarely reported but has occurred [18]. The person-to-person transmission of *C. psittaci* infection has long been suspected [47,48], but has only been documented over the past decade. In a 2013 outbreak in Sweden, one severely ill individual transmitted psittacosis to 10 others, including family members, hospital patients, and hospital staff [49]. A recent study reported five global examples of human-to-human psittacosis transmission, including a recent outbreak in China [50] that showed not only human-to-human transmission between infected cases and close contacts, but also secondary and tertiary transmission [51].

The incubation period for psittacosis averages 5–14 days but can last up to 39 days. Most who develop symptoms will do so within 5–14 days of exposure, with symptoms similar to mild community-acquired pneumonia (CAP). Psittacosis often manifests in young or middle-aged individuals as sudden onset fever, severe headache, and dry cough, although asymptomatic infection is possible [52]. Other common symptoms include fatigue, shortness of breath, chills, and myalgia [53]. Photophobia is often associated with a frequently severe headache. Pharyngitis, diarrhea, and impaired mental status are less common but significant symptoms. Diarrhea affects up to a quarter of patients and is often moderate but may be severe [54]. Other rarer signs and symptoms include pulse-temperature dissociation (fever without increased pulse rate), splenomegaly, and rash, though less frequently. More serious disease may initially manifest as atypical pneumonia. Neurologic symptoms are also rare but range from encephalitis to other central effects, such as cerebellar disturbance, transverse myelitis, intra-cranial hypertension and cranial neuropathies [55]. Dermatologic manifestations include a facial macular rash known as “Horder spots” [56].

Even with treatment, patients with psittacosis may have complications in a variety of organ systems. While uncommon, some can be serious. These include cardiac involvement, including endocarditis and myocarditis [57], pulmonary disease (e.g., respiratory failure), renal disease (e.g., acute tubular necrosis, acute tubulointerstitial nephritis, and acute proliferative glomerulonephritis), liver disease (e.g., icteric hepatitis, nodules, granulomas) [58], hematologic complications (e.g., hemolytic anemia, acute thrombocytopenic purpura, pancytopenia, and thrombotic thrombocytopenic purpura), neurologic disease (e.g., encephalitis, meningitis, and intracranial hypertension) [59], reactive arthritis [60], and cutaneous disease (e.g., erythema nodosum, erythema multiforme, erythema marginatum, and panniculitis) [1]. Severe psittacosis respiratory illness requires hospital-based ventilation and monitoring [61]. Although rare, psittacosis has occurred among organ transplant recipients [62]. While also uncommon, human gestational psittacosis may occur, and is associated with severe pneumonia and miscarriage. A review of 23 gestational psittacosis cases found an 83% fetal mortality rate and a 9% maternal mortality rate [63].

Psittacosis may be fatal if untreated [64]. Life-threatening *C. psittaci* endocarditis has been documented [65]. Human infections mostly occur as community-acquired pneumonia (CAP). A classical study of a bird-to-human transmission outbreak showed that of 38 individuals having an active immune response to *C. psittaci*, only 3 had sought medical attention [66]. Those infected with psittacosis and are only mildly symptomatic may not seek medical care [67].

## 6. Diagnostics

The differential diagnosis of psittacosis is usually that of atypical pneumonia, and includes the following: bacterial pneumonia; brucellosis; Chlamydia pneumonias; fungal pneumonias; infective endocarditis; Legionnaires Disease; *Mycoplasma pneumonia*; Q Fever; tuberculosis; tularemia; typhoid fever; and viral pneumonias [68].

Since most cases of psittacosis are mild, a definitive diagnosis is often not made. Some laboratory tests are difficult, and are often only available at specialized laboratories. Therefore, patients are often treated presumptively based on exposure history and clinical signs and symptoms. Nonetheless, the importance of *C. psittaci* infection as a cause of CAP is likely underestimated. A review of the literature from 1986 to 2015 using only studies of ≥100 patients revealed that *C. psittaci* was the causative pathogen in 1.03% of all CAP cases from the combined studies. For burden of disease estimates, it is a reasonable assumption that 1% of incident cases of CAP are caused by psittacosis [69].

Confirmation should certainly be made if the patient is at high risk of severe disease, fails to respond to appropriate treatment, or complications develop. The following diagnostic tests may be helpful in narrowing the differential diagnosis list given the disease presentation, coupled with a history of zoonotic contact: [70]

- Chest radiograph may demonstrate lobar or lobular pneumonia;
- Liver function tests may be slightly elevated;
- The erythrocyte sedimentation rate (ESR) may be elevated;
- Urinalysis may show mild proteinuria (<3500 mg/d).

Due to non-specific signs during psittacosis, the early detection of psittacosis infection and differentiation from hypersensitivity pneumonitis may be difficult. Cell culture and ELISA were once the ‘gold standard’ for *C. psittaci* detection, with poor results received from real-time polymerase chain reaction (PCR) analysis [71]. PCR has been used experimentally, and metagenomic sequencing data is being used more frequently for definitive diagnosis [72]. Today, commonly used laboratory techniques for positive pathogen identification include considerably improved PCR and serology [73]. While serologic testing is most often used, PCR has since evolved such that PCR testing is recommended for detecting the presence of *C. psittaci* in the lower respiratory tract and from other clinical specimens [74].

Serological tests used for *C. psittaci* detection include complement binding reactions, ELISA, immunofluorescence tests, and immuno-peroxidase tests. Serological testing, whether via the complement fixation or anti-Chlamydia microimmunofluorescence (MIF) assay, is known to evince cross-reactivity between the different species [75]. Both acute and convalescent samples are required for serological confirmation. There is a need for serologic *C. psittaci*-specific testing methods with good specificity and sensitivity that does not require convalescent serum sampling [76].

Human psittacosis (presenting as pneumonia) with a diagnosis based on clinical findings may be confirmed by PCR despite negative serological testing. The increased use of PCR for the early diagnosis of human psittacosis and the early initiation of correct antibiotic treatment has been recommended to reduce psittacosis morbidity and mortality [77]. Similar to PCR, metagenomic data from next generation sequencing (mNGS) has higher sensitivity and is faster than culture [53]. Metagenomics data has successfully been used to identify *C. psittaci* cases within 24 h [78]. Given the potential ability to find evidence of *C. psittaci* infection from NGS versus a negative serological test, NGS use has been referred to as ‘hypothesis-free pathogen detection’ [79].

To date, 15 genotypes of *C. psittaci* have been identified [80]. Infection with different *C. psittaci* strains can present with different clinical features. A case study of patients infected with *C. psittaci* strains SZ18-2 and SZ15 claimed that each patient demonstrated different clinical manifestations [81]. Such bacterial strain differences may also be rapidly detected with mNGS.

## 7. Treatment Options and Outcomes

The psittacosis mortality rate today is about 20% without treatment and as low as 1% with timely intervention. It is notable that psittacosis mortality was 50% in an outbreak in London in 1930 [3]. In the absence of appropriate and effective antimicrobial treatment, this disease’s considerable impact could reoccur.

Human psittacosis is effectively treated with doxycycline and tetracycline for 10–14 days, and as long as 21 days. For those whom tetracycline is contraindicated (pregnant woman and children <8 years) azithromycin and erythromycin are often used [82]. Fluoroquinolones are also active against *C. psittaci* infections but less than tetracyclines and macrolides [83]. With treatment, symptoms begin to regress after 24 to 48 h. Relapses have been known to occur. Severely ill patients require intravenous treatment with doxycycline hyclate [84]. Effective antimicrobial therapies have significantly contributed to the decreasing numbers of psittacosis cases. However, quinolones used to treat chlamydia infections have resulted in treatment failure [64]. Notably, *Chlamydia* can be induced through antimicrobial stimuli to undergo a temporary interruption in their replication cycle, entering into persistence, i.e., a viable but non-cultivable state. The regulatory mechanisms of *Chlamydia* persistence are unknown [85].

## 8. Potential for Use as a Bioweapon

As described, above, China, the former Soviet Union, and the US all explored *C. psittaci* use as a biological weapon. Infection among laboratory technicians at Fort Detrick through the inadvertent aerosolization of *C. psittaci* occurred in 1945 and 1961 [86]. At that time, *C. psittaci* was considered to be a virus due in part to the lack of electron microscopy and culture methods similar to those used in growing viruses [87].

Chlamydia may be genetically manipulated. The genetic manipulation of *C. muridarum* and *C. trachomatis* has been conducted using chlamydial plasmids [88]. Recently, a *C. pneumoniae* isolate from an equine source has been genetically altered to a create stable transformant using a plasmid shuttle vector system [89]. In the last year, *C. psittaci* has been genetically transformed using a plasmid shuttle vector adapted from *C. trachomatis* research containing a gene induction system, to produce stable *C. psittaci* transformants [90]. This work successfully inserted a gene for green fluorescent protein (GFP) that was expressed by stable *C. psittaci* transformants. However, these methods could also be used to insert other genes, including those promoting antimicrobial resistance. While there are inherent difficulties in the genetic manipulation of obligate intracellular bacteria such as Chlamydia, it is noteworthy that ongoing refinement of novel genetic tools continues at a rapid pace. These future improvements presently include the potential development of counterselectable markers to replace genes at desired sites in the chlamydial chromosome (now possible with the obligate intracellular bacterial pathogen *Coxiella burnetii*) [91], saturation mutagenesis enabling transposon-insertion sequencing [92], and the creation of synthetic riboswitches to control gene expression in Chlamydia [93,94].

Persistent infections of *C. psittaci* are thought to be associated with antimicrobial resistance. Cellular characteristics of this persistence includes the consistent downregulation of membrane proteins, chlamydial sigma factors, cell division protein, and reticulate body–elementary body differentiation proteins from 24 h post infection onward [95]. These molecular features then present another potential avenue for the genetic manipulation of *C. psittaci* to be able to create enhanced antimicrobial resistance.

The clinical and environmental recognition of *C. psittaci* is underappreciated worldwide, and therefore, the true burden is unknown. A 2021 metanalysis describes the global overall prevalence of chlamydial infections in birds as 20% and has been relatively consistent since 2012 [96]. Human cases in the US are now rarely reported, with less than 10 cases per year reported since 2010 but with occasional multistate outbreaks from poultry farms and processing plants, such as one that occurred in 2018 with 13 cases [97]. In 1978, there were 160 psittacosis cases reported from 33 states, and 116 cases reported from 25 states in 1979 [4]. Physicians’ awareness level of human psittacosis is low, and the disease is often undiagnosed [98,99]. The empiric antimicrobial treatment of those ill with psittacosis may mask the true magnitude of the disease [100]. This would become more apparent with antimicrobial treatment failure due to antibiotic-resistant strains or with severe illness [101]. The global misdiagnosis rate of psittacosis has been estimated at 50–80% [102,103]. Human psittacosis is not a nationally notifiable disease in some large at-risk nations, notably China and India. Further, some US states do not require the clinical reporting of psittacosis [104,105].

## 9. Conclusions

The CDC Category B agent *C. psittaci* is underappreciated and psittacosis has the potential for human-to-human spread. Psittacosis is often clinically undiagnosed, and the potential for protean disease manifestation exists. Unfamiliarity with complexities and difficulties encountered in the diagnostic testing and laboratory confirmation of psittacosis may enhance the lack of disease recognition. *C. psittaci*’s developmental cycle and immune evasion capacity contribute to its pathogenicity. Even should a clinical index of suspicion exist for this infection, there is an absence of required psittacosis disease reporting in many states and nations. Public health authorities may therefore be unaware of disease clusters that would otherwise initiate a more rapid public health response. Given the increasing potential for the genetic manipulation of Chlamydia that can be applied to *C. psittaci* to enhance infectivity, immune evasion, and antimicrobial resistance, the promotion of disease recognition, improved laboratory testing methods and public health awareness of psittacosis disease are strongly recommended.

## Data Availability

Not applicable.
